# Reactive Oxygen and Nitrogen Species in Plants

**DOI:** 10.3390/antiox13101232

**Published:** 2024-10-14

**Authors:** Francisco J Corpas, José M. Palma

**Affiliations:** Group of Antioxidants, Free Radicals and Nitric Oxide in Biotechnology, Food and Agriculture, Department of Stress, Development and Signaling in Plants, Estación Experimental del Zaidín, Spanish National Research Council (CSIC), C/Profesor Albareda 1, 18008 Granada, Spain; josemanuel.palma@eez.csic.es

Reactive oxygen and nitrogen species (ROS and RNS) include two families of molecules that, in recent years, have been shown to be involved in a wide range of biological functions, such as seed and pollen germination, the development and regulation of root architecture, stomatal movement, senescence, flowering, and fruit formation and ripening [1,2,3,4]. Additionally, ROS and RNS metabolism plays a crucial role in the response mechanisms to both abiotic and biotic environmental stresses [5]. These molecules are particularly significant because, at low concentrations, some of them possess signaling properties [6,7]. However, when ROS and RNS are overproduced and not properly regulated, they can induce nitro-oxidative stress, which may ultimately lead to cell death.

This Special Issue comprises eight manuscripts, seven of which are original research articles, while one is a comprehensive review. These research articles explore a diverse range of topics, including investigations on seeds, roots, and seedlings under both abiotic and biotic stress, as well as studies focused on fruits. Collectively, these works provide a broad and in-depth perspective on the critical roles of ROS and RNS across various plant organs, offering valuable insights into their significance in plant biology.

Griffo et al. (2023) [8] focused on analyzing the ROS content in seeds to assess whether they can serve as indicators of seed quality. For this study, the authors used seeds from soybean (*Glycine max*), tomato (*Solanum lycopersicum*), and wheat (*Triticum aestivum*), which were subjected to hydropriming (soaking the seeds in water and then drying them to improve germination) and further heat-shock treatment. ROS content was evaluated using the probes dichloro-dihydro-fluorescein diacetate (DCFH-DA) and ferrous oxidation-xylenol orange (FOX-1). To correlate these ROS content data, additional analyses were conducted to evaluate the expression of genes encoding enzymes involved in ROS metabolism, including *RBOH* (*respiratory burst oxidase homolog* family), *catalase*, *SOD* (*superoxide dismutase*), and *APX* (*ascorbate peroxidase*). The data suggest that the probes DCFH-DA and FOX-1 could be useful tools for evaluating seed quality. Another study targeted the role of ROS metabolism in abiotic stress. Thus, Flores-Cáceres et al. (2023) [9] investigated the involvement of ethylene in early oxidative stress triggered by heavy metals (mercury and cadmium) in alfalfa (*Medicago sativa*) seedlings. This study used 1-methylcyclopropene (1-MCP), which inhibits ethylene perception and attenuates the oxidative stress associated with Hg and Cd. The findings revealed that ethylene modulates ROS content; the accumulation of small heat shock proteins (sHSPs); the activity of glutathione reductase, an enzyme which is involved in the regeneration of reduced glutathione (GSH); and the subsequent accumulation of phytochelatins. On the other hand, Machado et al. (2023) [10] investigated the antioxidant systems, including SOD, APX, and catalase, as well as the lipid peroxidation content—a marker of oxidative stress—in tomato plants under nitrogen and water deficits, either separately or simultaneously. The results indicate a stronger response under combined stresses.

In their study, Minibayeva et al., 2023 [11] used wheat (*T. aestivum*) seedlings to analyze the accumulation of nitric oxide (NO) and ROS in roots when NO donors and the polyamine spermine were applied. They observed that this accumulation affected the redox status, particularly in mitochondria, which then triggered polyamine-induced autophagy. Based on their findings, they proposed that autophagy serves as a mechanism to remove oxidized and damaged cellular components. Yan et al. (2023) [12] studied root-knot nematodes (RKNs) (*Meloidogyne* spp.), which cause significant damage to sweet potato (*Ipomoea batatas* Lam.), leading to substantial losses in yield and quality. Their analysis of resistant and susceptible sweet potato cultivars provided clear evidence that antioxidant enzymes such as catalase, SOD, and peroxidases (PODs) play a key role in controlling ROS levels during different stages of infection.

Two studies in this Special Issue focus on fruits. González-Gordo et al. (2023) [13] investigated the gene and biochemical characterization of class III peroxidases during pepper (*Capsicum annuum* L.) fruit ripening and under a NO-enriched atmosphere. They identified ten *CaPOD* genes that were differentially regulated during ripening and in response to exogenous NO application. At the biochemical level, four CaPOD isozymes were identified. In vitro analysis showed that peroxynitrite, NO donors, and reducing agents caused nearly a 100% inhibition of CaPOD IV, suggesting that NO modulates H_2_O_2_ metabolism during pepper fruit ripening through NO-derived post-translational modifications. In another fruit study, Yan et al. (2023) [14] focused on the identification and analysis of *SOD* genes in the fruit of *Akebia trifoliata*, commonly known as the “chocolate vine”. They identified 13 *SOD* genes, including two *FeSODs*, four *MnSODs*, and seven *CuZnSODs*, which were differentially regulated during fruit development and in response to various pathogens, highlighting the crucial role of these antioxidant enzymes.

The review by Roussos et al. (2023) [15] updates the role of H_2_O_2_ and NO in the development of adventitious roots (ARs), a key mechanism for adapting to adverse environmental conditions such as hypoxia, flooding, or mechanical wounding. Although auxins play a central role in AR formation, other phytohormones—including cytokinins, gibberellins, abscisic acid, ethylene, and polyamines—also contribute to an overall response. However, H_2_O_2_ and NO have gained increased relevance, particularly when applied exogenously, as they enhance auxin-induced AR formation.

Finally, we would like to acknowledge all the authors who contributed to this Special Issue, which offers valuable insights into ongoing advancements in the study of ROS and RNS in plants. For researchers interested in this area, we are pleased to announce that a second edition of this Special Issue is now open for submission (https://www.mdpi.com/journal/antioxidants/special_issues/1HH36J258S) (accessed on 19 September 2024), and we encourage them to participate.
